# The paradox of screening: Rural women's views on screening for postnatal depression

**DOI:** 10.1186/1471-2458-10-744

**Published:** 2010-12-01

**Authors:** Susan J Armstrong, Rhonda E Small

**Affiliations:** 1 Mother and Child Health Research, La Trobe University, 215 Franklin Street, Melbourne, Vic 3000, Australia

## Abstract

**Background:**

Universal screening for postnatal depression is currently being promoted in Australia to assist detection and treatment of affected women, yet debate continues internationally about the effectiveness of screening. One rural shire in Victoria has been screening all women for postnatal depression at maternal and child health checks for many years. This paper explores the views of women affected by this intervention.

**Methods:**

A postal survey was sent to an entire one year cohort of women resident in the shire and eligible for this program [n = 230]. Women were asked whether they recalled having been screened for postnatal depression and what their experience had been, including any referrals made as a result of screening. Women interested in providing additional information were invited to give a phone number for further contact. Twenty women were interviewed in-depth about their experiences. The interview sample was selected to include both depressed and non-depressed women living in town and on rural properties, who represented the range of circumstances of women living in the shire.

**Results:**

The return rate for the postal survey was 62% [n = 147/230]. Eighty-seven women indicated that they were interested in further contact, 80 of whom were able to be reached by telephone and 20 were interviewed in-depth. Women had diverse views and experiences of screening. The EPDS proved to be a barrier for some women, and a facilitator for others, in accessing support and referrals. The mediating factor appeared to be a trusting relationship with the nurse able to communicate her concern for the woman and offer support and referrals if required.

**Conclusions:**

Detection of maternal depression requires more than administration of a screening tool at a single time point. While this approach did work for some women, for others it actually made appropriate care and support more difficult. Rather, trained and empathic healthcare providers working in a coordinated primary care service should provide multiple and flexible opportunities for women to disclose and discuss their emotional health issues.

## Background

Postnatal depression affects one in seven women in the twelve months after birth [1,2]. Depressed mood can impact on infant development as well as the health and wellbeing of the mother [3]. One approach to early intervention has been to screen recent mothers for postnatal depression with a validated screening tool. The most commonly used of these is the Edinburgh Postnatal Depression Scale [EPDS] which is freely available at no cost to the user and is easy and quick to administer [4]. Although the EPDS has been widely used in a variety of settings and in a number of countries for over 20 years there is a lack of evidence about the optimum timing and frequency of screening and about its effectiveness as a first step in improving outcomes for women screened in primary care[5]. While some have questioned its use in routine clinical care [6], others have advocated screening as being the best approach to improving detection and treatment of affected women [7].

In Australia the National Perinatal Depression Initiative is currently recommending universal routine screening of all women antenatally and postnatally [8]. Given the differing views about what constitutes best practice it is important that women's experiences of screening for postnatal depression are investigated.

The Maternal and Child Health Service from which the screening program being studied was carried out is part of a universal network of services for families that are provided in the state of Victoria through local municipalities with a defined geographic coverage. In rural areas these are often referred to as "Shires" and usually have smaller numbers of residents than urban local government areas. The Shire that formed the basis of this study had a population of 28,000 and was substantially rural in character with a number of small towns, none having more than 5000 residents.

This paper is a follow up of an earlier audit of screening for postnatal depression in a rural shire in Gippsland, Australia, where maternal and child health nurses [MCHNs] have been screening all women for postnatal depression using the EPDS. This practice had been in place for ten years but not previously evaluated. The study and found that universal screening for postnatal depression in routine clinical care was hard to achieve and did not lead to increased detection of depression in women. Further, the results of referrals made as a result of screening were unclear due to a lack of feedback [9]. The reasons for this were complex and involved both practical and interpersonal factors. Subsequently, changes were made to the way data were collected and the program simplified. Despite these practice improvements, a second audit in 2005-2006 showed that detection rates for postnatal depression in the shire were still lower than expected from population studies.

This paper reports on the experiences of women who gave birth during the second audit year, and aims to bring their views and experience of screening for postnatal depression more clearly into focus.

Our specific research questions were:

• How did women experience being screened for postnatal depression?

• Did screening for postnatal depression assist early identification and treatment and support for women affected by depression in the postnatal year?

• What were the pathways and barriers to care for rural women?

## Methods

A multi method approach was employed to explore women's experiences of screening for postnatal depression and of the first postnatal year. This included a postal survey sent to a one year cohort of recent mothers, telephone contacts with women who indicated on the survey that they were interested in further contact and in-depth interviews with a purposive sample of those contacted by phone.

On behalf of the research team, the local shire sent a letter of explanation and a brief questionnaire to all women who had given birth in the shire between 1 April 2005 and 31 March 2006 and would therefore have been expected to be screened as part of the postnatal depression screening program. The postal survey was successfully piloted with recent mothers from other health areas where the EPDS was also being used.

### The postal survey

A short survey was mailed to all women who had given birth during the study period, see additional file 1: Postal survey. It consisted of eight questions with fixed response alternatives and extra space for women to add brief comments in their own words. A copy of the EPDS was included as a memory aid, but marked not to be completed. A stamped addressed envelope was included for their reply and a reminder sent out four weeks later.

Daily (Monday to Friday) postal services in the region are supplied by Australia Post to all residences either directly or via pick up from a nearby rural mail box. Any items that are not able to be delivered are returned to the sender. Only 4 postal surveys sent out were returned on this basis, giving assurance that 98% were able to be delivered.

We were interested in knowing whether women remembered being screened for postnatal depression and what their experience had been, including any referral or treatment that had occurred as a result of screening. We indicated that we wished to speak with a small number of women in more detail and asked women who were interested in further contact to provide a first name and phone number. The postal survey had the advantage of giving all eligible women the opportunity to participate, while the in-depth interviews enabled us to get more detailed descriptions of women's experiences.

The study was approved by the La Trobe University Human Research Ethics Committee.

### Telephone contacts

A decision was made to contact all women who had provided phone numbers to thank them for their offer and to describe the purpose of the in-depth interviews. We also needed more information about the women's individual circumstances and depression experiences in order to select a range of women for interview. A standard phone protocol was used and notes of the key points in the phone call were recorded. The contacts lasted from 5-40 minutes. The women were reminded about the postal survey and asked whether they had experienced any difficulties with low mood or depression in the year following the baby's birth. Depression was defined as low mood in excess of two weeks. Women were also asked about the number and ages of children living in the family and what supports had been most helpful. We were interested in the positives of becoming a mother as well as hearing about what had been stressful.

### In-depth Interviews

The women selected for interview were offered the choice of being interviewed in their home or at a community based setting. Information about the project and the benefits and risks of participation were explained and written consents were obtained prior to interview.

A topic guide was developed asking women about:

• Physical and emotional health during the first postnatal year and any other significant events;

• Who conducted screening, how this was explained, and their experience of screening;

• Results of screening, including any referrals made or offers of help;

• Whether they considered themselves to have been depressed, or if they had received a formal diagnosis of depression, and whether they agreed with this or not;

• Sources of help during the first postnatal year including formally provided services and the informal support of family and friends.

• Any suggestions about things that would help women and families during the first postnatal year.

We selected women to maximise diversity of experiences with screening and of depression, and different family circumstances (one or more children, single mothers, families with stepchildren). We were conscious of selection for geographic location including women living in small towns as well as those in more remote locations and on farms. All interviews were taped and subsequently transcribed verbatim. The interviews represent a fuller exploration of issues that had arisen in the audit, postal survey and telephone contacts.

## Results

### Key Findings from the postal survey

Two hundred and thirty women who had given birth in the study year, and were still resident in the Shire, were surveyed by postal questionnaire. A total of 147 women completed and returned this, giving a response fraction of 62%. Over half [n = 87] gave their phone number and indicated that they were interested in further contact. The surveys were sent out in April 2007 and the telephone contacts and interviews were completed by December 2007. The infant triggering the survey was at this time between 12 and 24 months old. Most women recalled being asked to complete the EPDS at least once, with 11% stating they had not been asked. Fewer than half (48%) recalled completing it twice, as was actually stipulated in the revised screening protocol.

When asked how they felt about filling out the EPDS, 65% of the women surveyed stated that they didn't mind, although only 27% ticked that they thought it could be helpful.

Twenty-nine women (20%) added comments in the space provided for this question. Many of the positive or neutral comments were directed towards screening in general, or for "other women":

*I could see how it *[EPDS] *would pick up underlying problems*.

*I think it is good as some people who have PND don't realise it, as did a friend of mine*.

I did fill one out with my first child and it felt good that I could tick a box and say out loud when I did have a problem.

Ten women made negative comments about being screened and these were more personal, saying they were embarrassed or "felt exposed", and included comments about the lack of privacy when screening was offered in the maternal and child health clinic:

*A bit embarrassed. It reminded me of being back at school and then when she walked me through the "answers" I felt a bit exposed*.

*I knew that it could be helpful, although the nurse read out the questions and entered the answers which made me feel uncomfortable and unable to answer honestly*.

Of the sixteen women not screened, the most common reason given was that the nurse did not ask (n = 13), while three women stated that it was because they did not attend the maternal and child health service on a regular basis.

*I know the nurse personally and she quickly brushed over the topic assuming because I was working I was OK*.

The nurse knows me well enough to use her clinical judgement. Always talked openly anyway. Probably felt I was OK. Maternal and child health nurse was present at all my three births.

Most women found the EPDS easy to understand, with 85% reporting that "the statements in it all make sense". Only 10% reported difficulties in understanding some parts or were not sure.

Slightly more than half of the women surveyed (55%) stated that completing the EPDS made it easier to talk to the nurse about their feelings, 31% said that it didn't, with 14% not sure. Fifty-three percent of respondents (78/147) recalled the nurse making suggestions after the EPDS had been completed. In most instances this consisted of discussion of relevant issues or provision of written material about postnatal depression. Twelve per cent reported referrals being made to specialist services, including sleep, settling or day stay programs, counselling or home visits by MCH nurses. Six percent recalled being referred to their GP. Forty-six percent of the women (36/78) found the suggestions helpful.

*Any suggestions are helpful after having a baby. Just talking to someone who listens helps*.

*She suggested that she was just a phone call away*.

*She was very helpful. My husband was with me at the time and we all openly discussed my feelings and talked it all over amongst us*.

Ten women [7%] indicated that suggestions made by the nurse did not help them. Some women either disagreed with the results of screening, or felt there was no need for any help:

*I did not need any help. My answers had to do with uncommon feelings of worry that last week. I didn't display any symptoms of PND, therefore no need to refer me elsewhere*.

Some women were disappointed that screening had not resulted in them getting help and support:

*I needed something but no suggestions. I went on to a sleep clinic, but by finding out details myself*.

Another was more despondent:

*I felt a lot more depressed than the Edinburgh showed. Apparently crying every couple of days wasn't enough to warrant any further help. It made me feel like I was still on my own dealing with it*.

### Key findings from the telephone contacts

Eighty-seven women gave phone numbers for further contact. Eighty of these were reached by telephone. The respondents included primiparous and multiparous women. Twenty-six percent (21/80) said that they had been depressed during the first postnatal year with one or more of their children. Women spoke about their EPDS score as being high or low or normal, none recalled the actual score.

Forty-one of the 80 women contacted did not perceive themselves as having had significant difficulties with mood or depression. These women reiterated that they did not mind being screened and thought it could be helpful for "other" women. Thirteen women perceived themselves as having been depressed and said they had been helped by the screening program by receiving appropriate support or referral. Another 13 expressed reservations about the screening process and commented that screening had not made it easier to talk to the nurse about their feelings. Finally, 13 of the women contacted by phone had not completed the EPDS at all.

It was evident that screening was perceived by some women as a hurdle they had to surmount:

*I didn't do all that well on "the test", but no follow up was carried out*.

*I didn't mind doing the screening test. I was relieved to find that I was "normal"*.

Another woman worried about "*getting a high score*". She reported that the nurse said to her, "you are high, but you are not quite there."

Women spoke about choosing how to answer the items on the EPDS, and whether to be "honest" or put on a more positive front.

*It would be easy to misrepresent how you feel. It is a day to day affair. You could choose to share how you were feeling or keep it to yourself. Some days you might just want to weigh the baby and go*.

*When you go to the infant welfare you put on a positive front and talk about good things that happen. It is hard to switch to a more reflective mood*.

*I didn't mind filling in the EPDS but I didn't elaborate too much. I never spoke to the nurse about how I was **really **feeling. *[her emphasis]

One woman, a farmer who was being treated for depression by her GP admitted to not completing the EPDS honestly:

I didn't find the MCHN very helpful. I didn't want to talk to her about how I was really feeling. I always took my husband with me when visiting the centre.

Most of the 13 women who said they were helped by the screening process felt that they had a good relationship with the nurse and that she was supportive:

*I found the EPDS helpful. My nurse was easy to talk to. I knew I had postnatal depression because I had had it before. She gave me the EPDS and sent me to the doctor. He recommended medication and counselling*.

*My nurse was always helpful. She referred me to my doctor, who sent me to see a counsellor because I didn't want to go on medication. The counselling worked*.

However an equal number found that the screening process did not make it easier to talk to the nurse about their feelings and most were clear that their responses on the EPDS depended in part on their relationship with the nurse:

[I] *was very clear that the form was not the answer ... you could choose to share how you were feeling or keep it to yourself*.

[The screening] *was a bit superficial. There wasn't much talk about why it was being done or anything. It could have been better just to have a talk*.

*Completed the EPDS. Didn't think it helped. *[I] *wanted to talk in more depth with the nurse who just brushed it off. For 12 months... I really struggled*.

Support was mentioned by all mothers as being important in the first postnatal year and this often came instead, from family members and friends.

*If I had a problem I would talk first to a friend or my partner*.

I mean I wouldn't talk to a stranger about something like that. I'd ask my sister first before any stranger.

*My best support was my mother's group. If I *[then] *had a problem I would ask my MCHN or doctor*.

### Key Findings from the in-depth Interviews

Twenty women were interviewed in-depth. They ranged in age from 23 to 40, five were first time mothers and 15 had between two and five children. Ten of the women lived in small rural towns of less than 5,000 people, but accessible to services and shops, whereas seven lived in more remote areas, more sparsely populated and without access to public transport, and three lived on farms or isolated rural properties. Fourteen reported that they had suffered from depression during the first postnatal year for periods ranging from two to eleven months. The other six women did not consider themselves to have been depressed, though each reported having some "down" days. Three women had a previous history of depression prior to becoming a mother. Those women who described themselves as depressed did not see themselves as ill. They attributed their depression to psycho-social causes, parenting stress and the disturbed sleep associated with caring for an infant.

### The EPDS as a "good springboard for conversation"

For some women completing the EPDS had proved to be a good springboard for discussion whether or not they had felt depressed:

I found the EPDS helpful. It enabled us to talk about things we otherwise might not have done. It was a good springboard for conversation. We went through each question and had a bit of a laugh... "Does having two hours of sleep a night count?"

*Because I had had depression before I knew that something was not right. Doing the EPDS just confirmed what I needed to be told anyway*.

*It gave me that window of opportunity to sort of say, "Look, I have been feeling a bit down," or whatever and in my case, I kept telling my nurse how well I'd been doing but I was really quite,...I sort of prepared myself for the worst, that maybe this wouldn't be OK, but it gave me the opportunity to actually talk about it and any concerns I had. So, I probably didn't pay that much attention to the actual test itself, but it was just that conversation*.

Of the women who felt that screening had not made it easier for them to talk to their nurse there was a universal view that completing the EPDS did not address their particular needs; and for those who considered themselves depressed, but were not identified as such through the screening, it did not provide the help they wanted.

*I knew that it was important for me to talk about how I was feeling but I found it hard to do. I did the survey *[EPDS] *and the survey came up as me being mediocre. It didn't for me feel how I felt. I was withdrawing from everything I cared about at home, not enjoying myself and just not happy in myself*.

Of the four women interviewed who had not been screened, three had been feeling depressed. Two had sought help from their GP and were given a diagnosis of postnatal depression and offered treatment. The other was neither identified nor helped, by either her nurse or her GP, although she saw both health care providers regularly:

*They assumed that because I was at work I was fine and there was a form that I was supposed to have filled in *[the EPDS]. *I think I remember that she didn't have any at the time and I can just remember her saying, "Oh, you are working, you are fine."*

*Then when I saw the doctor he said "now, how is everything going?" as they do. I said "so, so", and I thought come on, that is the opening, ask more. But he moved on and I didn't feel like pushing it any further. I don't know why*.

### Stigma: "why did she give it to *me*?"

When women were offered the EPDS by the MCHN at one of their regular consultations some felt stigmatised, suspicious of the motives for screening, and ashamed to take up the suggestions for help:

*It was useful, but you also think, "Well, why did they give it to me? They must think there is something wrong with me straight away"*.

*She did say that it was just a survey and she probably said to me that everybody fills them out, but I didn't think that everybody fills them out as much as me, and I suppose when you're not confident in yourself, if you feel a bit down then... Oh I just thought she was making me fill these out 'cause she thought I was going to hurt my baby or I'm a bit of a nut and I just used to cry in there*.

*I was ashamed to go to the day stay. Like it took a long time for my mother-in-law to convince me. Everybody tells you the good things: oh this beautiful baby. I dressed it and bathed it and it looked good. But nobody tells you that it is OK not to cope and ask for help. Well that was my feeling. If you go to day stay you've failed*.

Many women reported being very conscious of how the nurse might view them in response to their answers:

I can remember thinking about the questions, trying to be honest, like being aware that the questions are loaded....And trying to be honest and yes I was honest, because I remember putting something down and thinking oh will that categorise me as being someone who has got postnatal depression or whatever?

### Timing and responsiveness

For some women the MCHN screening consultation had not been a suitable time or place to disclose their concerns about themselves:

*The nurse was just lovely but it wasn't really something that we could discuss that much because the baby was more the focus. He was really a very, very unwell baby, very colicky. So that was the focus and she said "Look, you have scored pretty badly on this thing," She didn't say badly but I can't remember whether it was high or low and she said, "You probably should see your doctor." And I sort of went "Oh, yes." And that was at about three months and then at about four months I started getting really bad thoughts, like "Oh, there's the toaster, plugged in." and "Oh, there's a knife." and "You can stick a knife in a toaster." And I thought "Oh yeah, they're pretty abnormal thoughts" I usually have a pretty level sort of head. I thought "That's pretty bizarre." And then I started getting all the hallucinations as well, which I think was sleep deprivation as well, I said "No, I'd better go to see someone." I went and saw the doctor. He was really good*.

Some noted that their mood on the day could affect their EPDS score:

*I just did a lot of self-reflection if that makes sense. Um and every-day this could change, that's the thing yeah. Some days I could have filled this thing up and it would have been straight to the doctor*.

Referring to the instruction on the EPDS to consider feelings over the past seven days, some women felt that a week was not enough time to give a real sense of how they were feeling. One woman suggested that a month would be better.

### The EPDS as a "test"

Comments during the telephone contacts that the EPDS felt like a "test" were further elucidated in some of the in-depth interviews. For some women the EPDS was something they clearly felt they needed to "pass", either to prove they were normal, or conversely, to qualify for help.

And, um, then I think when the survey came back and said I was borderline, I thought "Hmm. OK, well all right. How bad do you have to be before you...before you are classified as bad? To get to the point where you are wanting to hurt yourself?" There's no way in hell I want to get that bad.

One woman reported a similar understanding of the process, but with a more positive outcome:

*At the seven week visit I said to her "I think I am depressed." She said "Well, actually next week I was going to give it to you *[the EPDS]."

And so she gave me the test, and I completed it and she said, "It is showing that you are depressed." And I had the required number of answers to say that I was just over normal. She said "Look I can refer you to your GP who can refer you to a counsellor."

For some, the screening process was useful in validating their own assessment of their needs:

*Because I had had depression before I knew that something was not right. Doing the EPDS just confirmed what I needed to be told anyway*.

### A window of opportunity to talk

Women who found the screening process valuable most commonly reported that what was important to them was the opportunity to talk freely with the nurse and that completing the EPDS acted as a starting point to this. A positive relationship with the nurse was central to this being successful:

*The health centre nurse - she has been wonderful to talk to, has been very supportive. She was the one that actually picked up on the postnatal depression, before I realised myself what was going on*.

## Discussion

This is an exploratory study that contains detailed accounts of women's experience of a screening program for postnatal depression and although the findings are specific to one rural shire in the State of Victoria, they illustrate a range of issues in women's experiences that are likely to be pertinent to program and policy development for implementation of maternal depression screening in communities more generally. The study shire had been screening women for ten years but this program had not previously been evaluated and women's experiences with screening had not been assessed. Mitchell and Coyne in an article on issues of screening for postnatal depression in routine care point to the need to evaluate programs in context [10]. This study provides such an approach and presents important information about women's experiences of screening, not previously available in Australia.

Sixty-two percent of the women sent surveys responded in writing. This response fraction is very similar to the response to postal questionnaires obtained in other community-based studies of women in the postnatal period in Australia [11,12]. Although it is possible that a response bias may have been present it is significant that the women who did respond were generally representative of the overall cohort in terms of their age, number of children and the proportion who had been screened and not screened.

The combination of a postal survey, telephone contacts and in-depth interviews is a strength of the study. The survey provided valuable initial information about women's views on being screened for postnatal depression. The phone contacts then helped us to select a group of women with different experiences, who could be interviewed in more depth, to develop a richer understanding of how women understood and experienced the screening process in the context of their lives as mothers.

The women in our study experienced screening with the EPDS in diverse ways. While for some women completing the EPDS provided a welcome chance to talk about how they were feeling, others were suspicious of why they were being asked and expressed dissatisfaction with the screening instrument itself.

Issues with lack of privacy for mothers being screened, lack of referral services for women needing further assessment and treatment, and inadequate understanding of the limitations of screening by both women and health care providers have all been reported in previous studies of the experience of screening [13,14]. These were also found in our study. A recent meta-analysis found that in the majority of studies the EPDS was acceptable to women and healthcare providers when administered in the home, with prior training in empathic listening skills for the health providers and appropriate responses to Item 10, when women disclosed thoughts of self harm [15]. Screening women at home would be hard to achieve in rural Australia without additional resources and funding, especially in the area of mental health services, which are significantly harder to access in rural areas [16].

Seeing the EPDS as a pass/fail test, with a score that qualified some women for help and not others was a barrier to support and treatment for some women. Women often did not understand what was intended by screening, or the consequences of scoring as probably depressed. Poole and Mason [17] reporting on women's views of screening in the UK noted that the women interpreted the EPDS score as fact, that a high score meant women were depressed and a low score meant they were not.

Some women in our survey reported that they were screened for postnatal depression by their GP and that this was more acceptable than being screened by the nurse, especially in this small country area where knowing the nurse personally could cause discomfort for some - although not for others.

An Australian study which reported on 479 women who had been screened with the EPDS concluded that 81% were comfortable with screening. However comfort was inversely related to EPDS score and discomfort with screening was associated with more negative open ended comments on the screening process [18]. Dennis, in a systematic review of postpartum depression and treatment preferences found that difficulty in talking about feelings, shame and perceived social pressures commonly prevented women from seeking help for depressed mood [19].

In our study, for women perceiving themselves as not depressed, screening seemed to serve as a reassurance, or was viewed as not especially relevant personally, but likely to be useful for "other mothers". Many saw completion of the EPDS as confirmation to the nurse that they were coping well. Indeed most women are not depressed after birth, with around one in seven women suffering from depression at this time.

Screening all women may not be the best use of limited health resources, and may divert resources from treating women who are depressed and need more responsive care [20]. A recent health cost analysis of screening for postnatal depression versus standard care concluded that screening was in fact *not *cost effective [21].

In our study, the stigma of being depressed or perceiving themselves as not coping was not made easier for some women by the fact that screening was universal, contrary to the beliefs of some [12]. The paradox of screening for postnatal depression is that while screening may seem to make sense intuitively, in practice it appears to cause most difficulty for women who *are *depressed, those whom screening is really designed to identify and help. Women in our study valued the support of family, friends and peers and the chance to talk to a health care provider about their concerns, but only if they identified them as helpful and supportive.

One woman offered advice as to how health professionals could help.

*Sometimes you feel more like a number, like you don't matter. If they could have just taken 5 minutes to chat with me one on one that would help. Asking for help is the hardest thing to do*.

## Conclusion

The views of women documented here demonstrate that a program of universal screening for postnatal depression in routine care was not universally seen to be helpful. For some, screening did provide a useful opportunity to talk about how they were feeling, while for others screening was viewed with suspicion and did not result in their receiving help. Flexible opportunities for women to disclose and discuss their emotional health issues with trained and empathic healthcare providers may lead to better outcomes than a routine system of screening with a pen and paper "test".

## Competing interests

The authors declare that they have no competing interests.

## Authors' contributions

SA conceived the research question, and developed the research design, conducted all the interviews and drafted the paper. RS contributed to the research design and re-drafting of the final paper. Both authors read and approved the final manuscript.

## Pre-publication history

The pre-publication history for this paper can be accessed here:

http://www.biomedcentral.com/1471-2458/10/744/prepub

## Supplementary Material

Additional file 1 **Postal survey**. Copy of postal survey sent to women in the study Click here for file
