# Supplementary material for: The paradox of screening: Rural women's views on screening for postnatal depression
Source: BMC Public Health. 2010 Dec 1;10:744. doi: 10.1186/1471-2458-10-744 (PMC3009649; doi:10.1186/1471-2458-10-744)
Supplement: Additional file 1 — Postal survey. Copy of postal survey sent to women in the study [file 1471-2458-10-744-S1.DOC]

### Women's Experience of Screening for Postnatal Depression

We are interested in finding out more about women's experience of screening for postnatal depression. Enclosed with this form is a copy of the Edinburgh Postnatal Depression Scale (EPDS) which is given to women in the Shire when they attend their local Maternal and Child Health Centre on two occasions, at two and eight months, after they have had a baby. Please do not fill it in now. It is only provided to remind you. We would like to ask you some questions about when (and if) you completed the EPDS since you had your baby in 2005. Please tick the box next to the statement that you agree with.

**1. Do you remember being asked by your Nurse to fill in the EPDS?**

□ Yes, at about two months after my baby’s birth.

□ Yes, at about eight months after my baby’s birth.

□ No, I can’t remember being asked.

**2. Did you actually go on to fill out the EPDS?**

□ Yes, I remember filling it out once.

□ Yes, I remember filling it out twice.

□ No, I didn’t fill it out at all.

**3. How did you feel about being asked to fill out the EPDS by the Nurse? ( If you can’t remember doing it, tell us how you think you would have felt.)**

□ I didn’t mind

□ I thought it could be helpful.

□ I couldn’t see the point to it.

□ Other: please explain… .........................................................................................................................................................................................................................................................................................................................................................................................................................................................

**4. Do you think the EPDS is easy to understand?**

□ Yes. The statements in it all make sense to me.

□ Some parts of it are hard to understand.

□ No, I think most of it is hard to understand.

□ Not sure.

**PLEASE TURN OVER-------------------------------🡪**

**5. If you didn’t fill out the EPDS why not?**

□ I didn’t go to the Maternal and Child Health Centre regularly.

□ The Nurse didn’t ask me to fill it out.

□ I was more concerned about the baby than myself.

□ I just didn’t want to answer the questions.

□ I felt fine and didn’t see the need to do it.

□ Other..please explain

…………………………………………………………………………………………………………

…………………………………………………………………………………………………………

…………………………………………………………………………………………………………

**If you do remember completing the EPDS, please answer the following questions:**

**6. Did filling out the EPDS make it easier for you to talk to the nurse about your feelings?**

□ Yes □ No □ Not sure

**7. Did the nurse make any of the following suggestions to you after you filled in the EPDS?**

□ She didn’t make any suggestions.

□ She suggested I should see my doctor

□ She gave me some written information about postnatal depression and where I could go for further help.

□ She suggested I go to a sleep and settling or day stay program.

□ She referred me to a counsellor

□ She arranged for a Nurse to visit me at home.

□ Any other suggestions she made…please describe

………………………………………………………………………………...…………………………………………………………………………………………………………...…………………………

………………………………………………………………………………...…………………………

**8. Were any of these suggestions helpful?**

□ Yes…please describe how

………………………………………………………………………………...…………………………………………………………………………………………………………...…………………………

………………………………………………………………………………...…………………………

□ No…please describe why not

………………………………………………………………………………...…………………………………………………………………………………………………………...…………………………

………………………………………………………………………………...…………………………

### We would like to talk to some women in more detail about their experiences in the first year after having a baby. We are interested in finding out both about things that may be stressful and also what services and supports (including family and friends) are helpful. If you are willing for us to contact you could you please write your first name and phone number in the space below. We would like to hear from you whether or not you were screened for postnatal depression.

### Name Telephone number

### ………………………. ………………………..

### The best time to contact me is...

⁯ Morning ⁯ Afternoon ⁯ Evening ⁯ Any time

### Your details are confidential and will not be passed on to anyone else. If you are able to help further we would very much appreciate it and we believe that it could help other mothers in the shire.

### Thank you very much for your time and attention to these questions.

### Please return this form by putting it in the enclosed envelope and sending it by mail.
